# Structure of a bacterial type III secretion system in contact with a host membrane *in situ*

**DOI:** 10.1038/ncomms10114

**Published:** 2015-12-11

**Authors:** Andrea Nans, Mikhail Kudryashev, Helen R. Saibil, Richard D. Hayward

**Affiliations:** 1 Department of Crystallography, Institute of Structural and Molecular Biology, Birkbeck College, Malet Street, London WC1E 7HX, UK; 2 Focal Area Infection Biology, Biozentrum, University of Basel, Klingelbergstrasse 50/70, CH-4056 Basel, Switzerland; 3 Center for Cellular Imaging and NanoAnalytics, Biozentrum, University of Basel, Mattenstrasse 26, CH-4058 Basel, Switzerland; 4 Institute of Structural and Molecular Biology, Birkbeck and University College London, Malet Street, London WC1E 7HX, UK

## Abstract

Many bacterial pathogens of animals and plants use a conserved type III secretion system (T3SS) to inject virulence effector proteins directly into eukaryotic cells to subvert host functions. Contact with host membranes is critical for T3SS activation, yet little is known about T3SS architecture in this state or the conformational changes that drive effector translocation. Here we use cryo-electron tomography and sub-tomogram averaging to derive the intact structure of the primordial *Chlamydia trachomatis* T3SS in the presence and absence of host membrane contact. Comparison of the averaged structures demonstrates a marked compaction of the basal body (4 nm) occurs when the needle tip contacts the host cell membrane. This compaction is coupled to a stabilization of the cytosolic sorting platform–ATPase. Our findings reveal the first structure of a bacterial T3SS from a major human pathogen engaged with a eukaryotic host, and reveal striking ‘pump-action' conformational changes that underpin effector injection.

## Abstract (web summary)

Bacterial type III secretion systems (T3SSs) inject virulence effector proteins into eukaryotic cells and are activated by host membrane contact. Here the authors report the *in situ* structure of the *Chlamydia trachomatis* T3SS in the presence or absence of host membrane, and observe compaction of the basal body embedded in the bacterial envelope.

## Graphical abstract

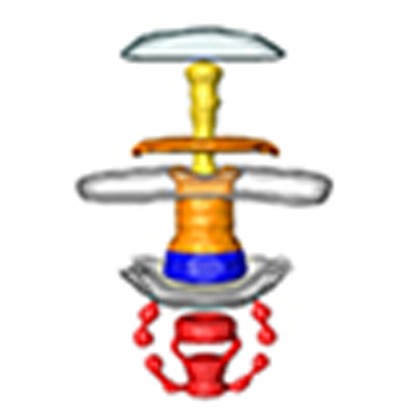

Bacterial type III secretion systems (T3SSs) are central to the virulence of a wide spectrum of medically important pathogens. T3SSs are conserved membrane-embedded nanomachines that deliver virulence effector proteins directly from the bacterial cytosol into the host cell^1^,^2^. Cryo-electron microscopy and X-ray crystallography studies of isolated T3SS core complexes and their components have characterized the basal body that spans the bacterial envelope^3^,^4^,^5^,^6^,^7^,^8^,^9^,^10^,^11^. This is formed of membrane-embedded oligomeric rings that are connected by a trans-periplasmic rod to a hollow needle through which unfolded effectors are secreted. Recent *in situ* studies using bacterial minicells identified an additional associated complex in the bacterial cytoplasm comprising a sorting platform and export ATPase^12^,^13^.

*Chlamydia trachomatis* is an obligate intracellular pathogen and the major bacterial cause of sexually transmitted disease worldwide^14^. Ocular infections cause blinding trachoma, which is recognized as a neglected tropical disease by the World Health Organization. The structure of the chlamydial T3SS complex has never been elucidated, but it is among the nearest phylogenetic relatives to the bacterial flagellum, suggesting that Chlamydiae harbour a primordial virulence-associated T3SS (refs 15, 16, 17). Unlike other Gram-negative bacteria where contiguous T3SS operons are located on a virulence plasmid or in distinct chromosomal pathogenicity islands, chlamydial genes encoding T3SS components are located in four distinct clusters dispersed across the entire genome. In addition, despite being immotile bacteria, Chlamydiae apparently harbour genes for flagellar components, encoding homologues of the inner membrane ring (*fliF*), a major component of the export apparatus (*flhA*), the ATPase (*fliI*) and a component of the sorting platform (*fliH*), although the function of these orphan proteins remain unknown^15^,^16^,^17^.

Wild-type chlamydial elementary bodies have dimensions favourable for cryo-electron tomography^18^ (300–400 nm diameter), which eliminates the requirement to genetically derive artificial minicells or the use of osmotic shock for *in situ* imaging. To investigate the structural changes that accompany host membrane contact and T3SS effector translocation, we have performed whole cell cryo-electron tomography^19^ and sub-tomogram averaging of the *C. trachomatis* T3SS in the presence or absence of host membrane contact. Sub-tomogram averages show a striking compaction of the basal body and structural stabilization of the sorting platform-ATPase complex upon T3SS needle contact with a host membrane. These data provide insights into the physiologically relevant conformational changes in the T3SS *in situ* following host membrane contact and subsequent activation of secretion.

## Results

### *In situ* structure of the host-free *C. trachomatis* T3SS

**Figure 1:**
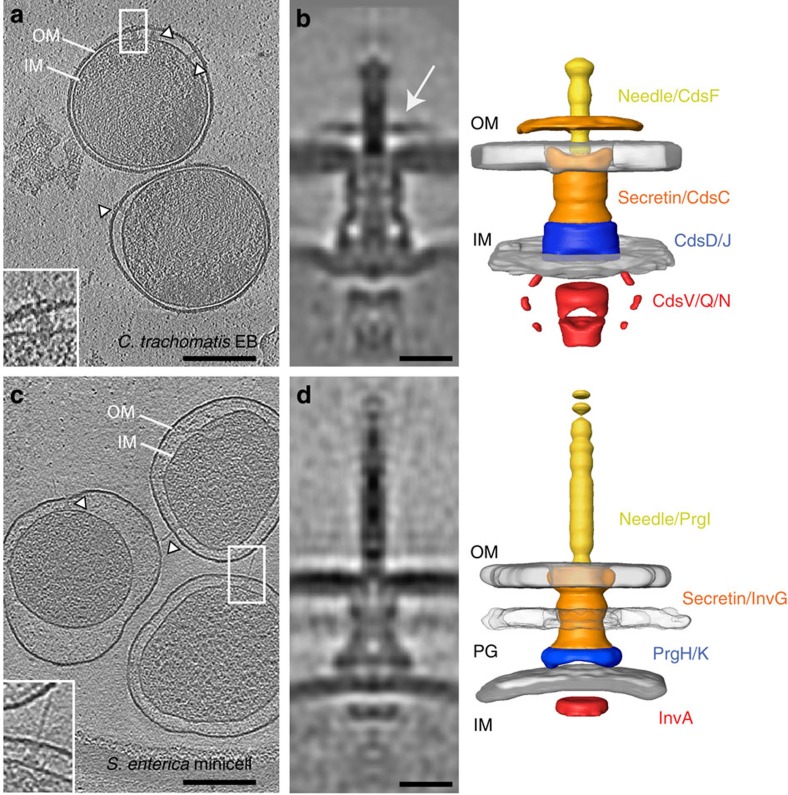
*In situ* sub-tomogram averages of host-free *Chlamydia* and *Salmonella* T3SS. Central slices (10 nm thick) from denoised cryo-electron tomograms of plunge-frozen *C. trachomatis* LGV2 elementary bodies (**a**) and *S. enterica* minicells (**c**). Outer (OM) and inner membranes (IM), peptidoglycan layer (PG) and type III secretion systems (white arrowheads and magnified in the lower-left corner) are indicated. Central slices through sub-tomogram averages of the host-free T3SS from *C. trachomatis* (**b**) and *S. enterica* (**d**). Individual components are labelled in the corresponding three-dimensional surface rendering (right). Novel ring that surrounds the *Chlamydia* needle is highlighted (white arrow). Scale bars, (**a**,**c**) 200 nm; (**b**,**d**) 15 nm.

Initially, we imaged intact *C. trachomatis* elementary bodies by cryo-electron tomography in the absence of host cells (Fig. 1a). Elementary bodies are inherently polarized and one bacterial hemisphere is characterized by a pronounced widening of the periplasmic space that accommodates a semi-ordered array of T3SS complexes^18^. Of 906 T3SS complexes initially selected, we conducted sub-tomogram averaging using a subset of 515 to generate an *in situ* structure at a resolution up to 3.3 nm (Fig. 1b and Supplementary Fig. 1a). The overall architecture of the chlamydial T3SS is distinct from every other T3SS observed to date either in the bacterial envelope *in situ* by tomography or in isolation by single-particle analysis (Supplementary Fig. 1b)^3^,^4^,^5^,^12^,^20^,^21^. The *C. trachomatis* basal body is more elongated with a pronounced convex curvature, a length of 34 nm and a diameter that ranges from 14 to 20 nm. Nevertheless, densities corresponding to the core T3SS components, the outer membrane secretin (CdsC) and inner membrane oligomeric rings (CdsD and CdsJ) are discernible. A novel ring surrounds the needle on the face of the outer membrane, which likely corresponds to an additional secretin domain, as no pilotin or additional predicted outer membrane proteins are present within the operons encoding *C. trachomatis* T3SS components. Indeed, phylogenetics shows that CdsC is atypical as it clusters with type II secretins of filamentous phage^15^. At 921 residues it is significantly larger than all other T3SS secretins (for example, *Shigella* MxiD and *Yersinia* YscC at 566 and 607 residues, respectively). The additional residues include a unique N-terminal 250-residue hydrophilic domain and type II secretin-specific insertions within the N-domains and secretin homology domain (Supplementary Fig. 2)^15^. There is precedent for type II secretins to extend beyond the confines of the outer membrane, supporting the unusual structure of CdsC among T3SS secretins^22^.

The chamber within the basal body is well resolved, with a clear view of the cup and socket that anchor to the inner rod proteins^23^. No peptidoglycan was observed, in agreement with labelling studies^24^. The inner membrane exhibits a concave deformation at the point of basal body integration, while the rigid outer membrane remains undistorted. A density characteristic of the cytoplasmic domain of the export apparatus (CdsV) is present on the cytoplasmic face of the inner membrane proximal to the basal body^10^. As recently visualized in *Shigella flexneri*^12^, the cytoplasmic sorting platform (CdsQ)–ATPase (CdsN) complex is also evident, although this is less distinct in comparison to the remainder of the structure, suggesting an increased disorder. At the outer membrane, a hollow needle 28 nm in length with a well-resolved needle-tip complex extends from the basal body (Fig. 1b). Given the striking differences in the architecture of the *C. trachomatis* needle, the basal body and the envelope at the site of integration, we verified the *in situ* structure of the archetypal SPI-1 T3SS from *Salmonella enterica* using minicells. The architectural differences between the *Salmonella* and *Chlamydia* T3SSs are clear even from the raw tomogram slices (Fig. 1c), and sub-tomogram averaging of the *Salmonella* T3SS using an identical image-processing protocol recapitulated a complex with expected features at 4.5 nm resolution, including the peptidoglycan layer (Fig. 1d and Supplementary Fig. 1a). Interestingly, the chlamydial needle is only half the length of that in *Salmonella* (∼60 nm), reflective of the unusual outer membrane structure in *Chlamydia*. The short needle length is entirely consistent with the presence of shorter, deep-rough chlamydial lipopolysaccharide, which contains only lipid A and three terminal Kdo residues^25^,^26^. Needle length is tightly regulated and linearly correlates with the size of the tape measure protein (SctP)^27^. Accordingly, the 283-residue CdsP from *Chlamydia* spp. is shorter than *Salmonella* InvJ (336 residues; Supplementary Fig. 3). These data illustrate that while sharing the major hallmarks of other systems, the architecture of the primordial chlamydial T3SS differs significantly from those studied previously.

### *In situ* structure of the host-contact *C. trachomatis* T3SS

**Figure 2:**
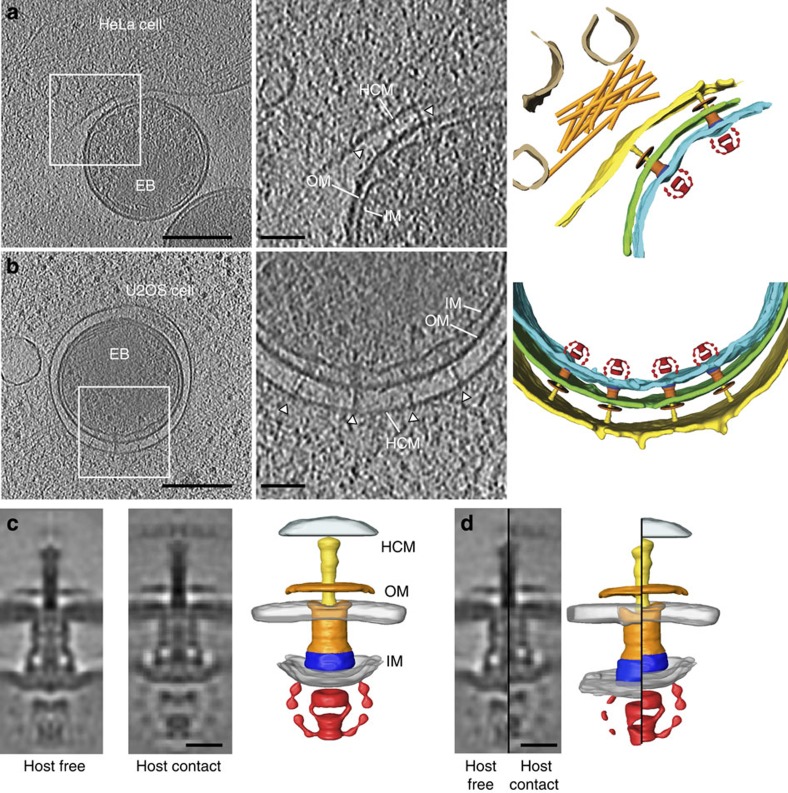
*In situ* structure of host-contact *Chlamydia* T3SS. (**a**,**b**) Central slices (10 nm thick) through denoised cryo-electron tomograms of an elementary body bound to a HeLa cell plasma membrane via a type III secretion system (**a**) and within a vacuole of a U2OS cell (**b**). Magnified views and the corresponding three-dimensional (3D) surface renderings are shown on the right. Outer (OM, green), inner (IM, cyan) and host membranes (HCM, yellow) are indicated along with type III secretion systems (white arrowheads). Actin filaments (orange) and intracellular vesicles (brown) are present in the host cell cytosol. (**c**) Central slice through the sub-tomogram average of the host-free (left) and host-contact (middle) *C. trachomatis* T3SS. In the corresponding 3D surface rendering (right), secretin (orange), inner membrane ring (blue), sorting platform, export apparatus and ATPase (red) are highlighted. Host cell membrane (light blue) is also visualized. (**d**) Side-by-side split-view comparison of host-free and host-contact *Chlamydia* T3SSs and the corresponding surface renderings. Scale bars, (**a**,**b**) 200 nm (left) and 50 nm (right) and (**c**,**d**) 15 nm.

*Chlamydia* T3SSs can frequently be captured in contact with host membranes at the cell periphery during internalization and early vacuole development^18^. To derive the *in situ* structure of the chlamydial T3SS in contact with host membranes, we visualized *C. trachomatis* elementary bodies early during entry into cultured cells by cryo-electron tomography, either in association with the host plasma membrane (Fig. 2a) or within early vacuoles (Fig. 2b). In both states, multiple T3SS–host membrane contacts were present with an average spacing between the bacterial outer membrane and host membrane of 33±4.0 nm (Supplementary Fig. 4a). In contrast, the average distance at nonspecific bacteria–host cell adhesion sites was 20±3.0 nm, illustrating that T3SS-mediated contacts are defined junctions determined by the length of the needle. The number and spacing of the *Chlamydia* T3SS within the polarized array remained unchanged on host cell contact (57±13 nm host free, *n*=58 versus 54±9 nm host contact, *n*=60), unlike *Yersinia* that forms clusters of T3SSs incorporating new assemblies on activation^28^.

Of 279 T3SS–host complexes initially selected, we conducted sub-tomogram averaging using a subset of 196 to generate an *in situ* structure of the T3SS in contact with the host cell, up to 3.8 nm resolution (Fig. 2c,d and Supplementary Fig. 1a). We observed several significant differences between host-free and host-contact structures. Strikingly, the length of the basal body in the host-contact state is ∼4–5 nm shorter than that in the host-free T3SS (29 versus 34 nm). This compaction is accompanied by a subtle widening in the density corresponding to the cytosolic domains of the export apparatus, although the outer diameters and the overall architecture of the complex remain similar (Fig. 2c,d and Supplementary Fig. 4b). The length reduction occurs uniformly throughout the densities corresponding to the inner membrane ring (CdsD) and the secretin (CdsC), suggesting that the interacting periplasmic domains of these components are involved in basal body contraction. This is consistent with the basal bodies of the *Y. enterocolitica* T3SS that also exhibit inherent flexibility in the host-free state, whereby the periplasmic N-terminal secretin domains confer this plasticity^20^,^29^. Similarly, the C-terminal periplasmic domains of *Yersinia* YscD, the major component of the inner membrane ring, adopt extended or compact conformations in isolation^20^. In contrast to the *Shigella* and *Salmonella* inner membrane ring proteins^9^,^12^, CdsD is likely fully extended. The CdsD cytoplasmic domain is considerably larger than its homologues, as it contains an additional FHA domain (Supplementary Fig. 5). This is consistent with the CdsD N-terminus protruding further into the cytosol than in other characterized T3SSs.

In addition to this contact-associated compression of the basal body, the densities of the cytoplasmic sorting platform and the ATPase are considerably more pronounced, despite the lower resolution of the average structure (Fig. 2d and Supplementary Movie 1). Similar to the *S. flexneri* T3SS (ref. 12), the sorting platform exhibits six-fold symmetry, but the resolution of our sub-tomogram average is too low to unequivocally determine the symmetry of the basal body (Supplementary Fig. 6). Our data suggest that the host-free sorting platform–ATPase complex is either transiently associated with the basal body or adopts variable orientations, thus causing it to be poorly resolved in the averaged structure. Contact with a host membrane and subsequent activation of type III secretion leads to a structural stabilization in the C-ring sorting platform, yielding sharper and better-defined features in the structure. However, the dimensions of the sorting platform–ATPase complex are similar in the two states, suggesting there is no compaction of the sorting platform components during host membrane contact (Supplementary Fig. 4b).

Consistent with the constant distance between the bacterial outer membrane and the host membrane at T3SS-mediated contacts (Supplementary Fig. 4a), the host cell membrane is also resolved in the sub-tomogram average despite being excluded from the alignment mask (Fig. 2d). A pore-like density proximal to the needle tip was evident in the host cell membrane, implying the assembly of a continuum between the bacterial cytosol, T3SS complex and the host cytosol. However, although inherently suited to cryo-electron tomography, Chlamydiae remain genetically intractable. Therefore, this intriguing structure cannot unequivocally be assigned as the T3SS translocon in the absence of bacterial mutants lacking the predicted CopB/CopD components^30^,^31^,^32^. While the inner membrane deformation is apparently retained during stabilization of the sorting platform–ATPase complex, the outer membrane adopts an increased curvature, indicating that even the highly rigid elementary body surface undergoes local adjustments on needle–host contact. Indeed, the novel ring that surrounds the emergent needle on the bacterial surface might facilitate such flexibility (Supplementary Movie 1).

### Structural heterogeneity of the *C. trachomatis* T3SS

**Figure 3:**
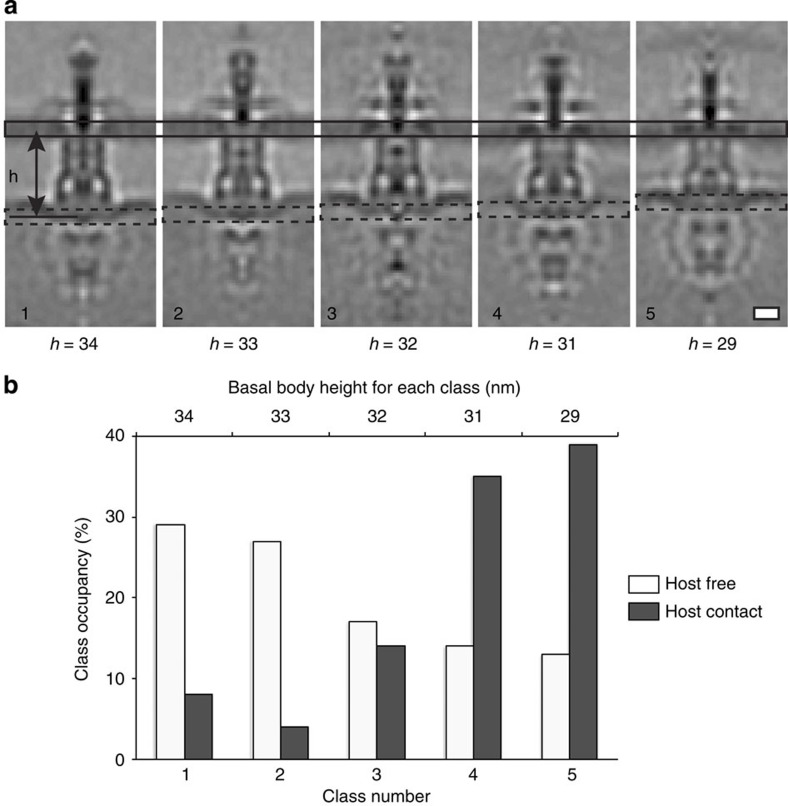
Structural variability of *Chlamydia* T3SS sub-tomograms by multi-reference alignment. (**a**) Central slices through five class averages of *Chlamydia* T3SSs generated by non-biased multi-reference alignment in Dynamo. The class averages are sorted by basal body height (*h*), which varies from 34 to 29 nm. (**b**) Distribution of host-free and host-contact *Chlamydia* T3SSs across the five classes depicted in **a**, sorted by basal body height. Host-contact T3SSs were mainly present in classes with short basal bodies with defined sorting platform–ATPase complexes (classes 4 and 5). Scale bar, 15 nm.

While the host-free and host-contact *Chlamydia* T3SS are structurally distinct, the individual complexes do not describe potential intermediate states involved in T3SS activation. Therefore, to quantify the extent of structural heterogeneity within the sub-tomograms, we classified the chlamydial T3SS data set into five or ten classes by multi-reference alignment (MRA). Alignment for MRA was conducted using a bottle-shaped mask that included the basal body, inner membrane and sorting platform–ATPase complex. As would be expected due to the limited number of sub-tomograms per class, the resulting class averages have a lower resolution when compared with the structures of host-free and host-contact T3SS that were generated from the respective complete data sets. Nevertheless, the resulting class averages still revealed a continuum of basal body lengths, ranging from 29 to 34 nm with differing sorting platform–ATPase complex structures (Fig. 3 and Supplementary Fig. 7). MRA classification was not biased by the missing wedge of information, as there is no correlation between particle orientation and class assignment (Supplementary Fig. 7). Analysis of the class occupancy showed that the majority of host-contact T3SSs partitioned into classes with the shorter basal bodies, whereas host-free T3SSs distributed into the classes exhibiting longer basal bodies with a minority classifying as the short form. Extracellular T3SSs attached to the plasma membrane and T3SSs within a vacuole equally contributed to the classes with short basal bodies, illustrating that there is no structural distinction between these two host-contact sub-states and that basal body compaction is likely not the result of physical constraints imposed by the vacuole. The structure of the cytosolic sorting platform–ATPase complex had a varied appearance across the classes containing longer basal bodies (heights from 32 to 34 nm). However, the associated density was significantly more pronounced in the two classes with the shorter basal bodies (heights from 29 to 31 nm), corresponding to the host-contact state and appearing clearest in a single class (29 nm) (Fig. 3). This suggests that active T3SSs may adopt different states on host membrane contact with defined basal body lengths and different sorting platform stabilities.

## Discussion

**Figure 4:**
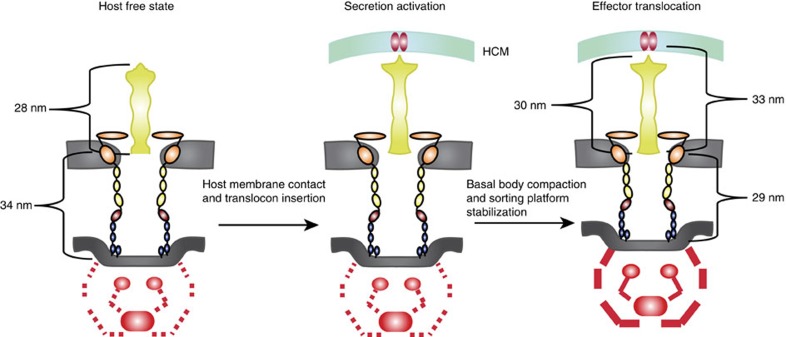
Model for activation of type III secretion. (Left) The *Chlamydia* type III secretion system basal body is composed of inner membrane rings (CdsD and CdsJ; blue) and together with the outer membrane protein secretin (CdsC) form a multimeric channel spanning the entire periplasm. The *Chlamydia*-specific N-domain (dark red), classical N-domains (yellow) and secretin homology domain (orange) of CdsC are indicated. The multiple periplasmic domains of both the secretin and inner membrane rings are joined by linkers, which govern the flexibility and length adaption of the basal body. In the host-free state (left), the basal body exhibits a length of 34 nm with needle 28 nm long. The major component of the export apparatus (CdsV) and ATPase (CdsN) are present (red) but the sorting platform (CdsQ) is largely absent (red dashed lines; centre). On needle–host membrane contact, the translocon is inserted into the plasma membrane, an event associated with conformational changes or elongation in the needle and tip complex. Following activation (right), the basal body contracts 4 nm by compacting the periplasmic domains closer together. The sorting platform structurally stabilizes in the cytosol, proximal to the basal body, primed for translocation of effectors.

We have resolved the first *in situ* structure of a bacterial T3SS in contact with a eukaryotic host, revealing coordinated changes in the needle, basal body and cytoplasmic sorting–ATPase complex that accompany activation. Our data demonstrate that the T3SS truly behaves as a ‘molecular syringe', which contracts by compressing the periplasmic domains of the basal body components. Concomitantly, the sorting platform–ATPase complex becomes stabilized, primed to engage effector substrates for export (Fig. 4). The precise sequence of events underlying secretion activation has not been completely established, but current models suggest that the process is initiated when the needle tip complex senses the host^33^,^34^. The tip complex may then also function as an assembly template for the insertion of the translocon into the host membrane^35^,^36^,^37^. Subsequent conformational changes in the needle tip and the needle itself presumably allow signals to be transmitted distally to the bacterial cytosol^38^,^39^,^40^. Effectors destined for secretion are recruited to the sorting platform and unfolded before transfer through a channel within the basal body, the needle and finally across the host plasma membrane^13^,^41^.

We suggest that on host membrane contact the basal body favours the compact form and the sorting platform–ATPase complex adopts a more consistent orientation. These changes are accompanied by a subtle widening of the cytosolic aperture of the export apparatus. Thus, this may reflect a superstructural state that enables effector translocation across three membranes. Our sub-tomogram classification reveals a continuum of T3SS forms that favour extended or compact basal bodies in the cell-free and cell-contact states, respectively. Chlamydial elementary bodies present a polarized array of T3SSs to the host, but it is unlikely that all the individual needles on the bacterial surface are optimally positioned to actively engage the host cell surface, or are necessarily simultaneously activated, resulting in a mixed but morphologically biased population. Similarly, a proportion of the host-free T3SSs cohort exhibits compact basal bodies (<30%), reminiscent of the contact state. This could be attributed to the presence of artificial inducers such as serum- or cholesterol-containing membrane fragments in the culture medium, which can trigger type III secretion in chlamydial elementary bodies^42^. Indeed, analysis of *Yersinia* T3SSs in the host-free state revealed a similar distribution of extended and compact basal body conformations under Ca^2+^-depleted conditions, when secretion is activated in the absence of host cells^20^. Such dynamic temporal associations within the T3SS are further supported by recent quantitative fluorescence approaches showing that YscQ, a major component of the *Yersinia* sorting platform, exists both in stable association with the T3SS and dissociated in a cytosolic pool^43^. Interestingly, increased YscQ subunit exchange between the T3SS and cytosolic pool was observed when secretion was induced artificially, supporting the view that structural rearrangements may occur in the sorting platform on activation. By contrast, the analogous platform in related flagellar motors (FliG/M/N) is constitutively associated with the basal body as it is essential for transferring the torque to rotate the motor^44^. In virulence systems, the sorting platform–ATPase complex may be involved in activity-associated basal body contraction rather than continuous rotation.

In addition to providing important general insights into the structural transitions that accompany T3SS interaction with the host membrane and effector translocation, our analyses have revealed key structural distinctions between the chlamydial T3SS and those already extensively studied in other pathogens. In particular, while sharing distinguishing characteristics, the basal body architecture is surprisingly divergent from canonical examples, and the needle is associated with an additional ring-like structure on the outer membrane surface. Further studies of this system, expedited by emerging genetic techniques^45^,^46^, will enable the precise assignment of chlamydial T3SS components and advance the understanding of the evolution of virulence-associated T3SSs.

## Methods

### Reagents, cell culture and *Chlamydia* propagation

All cell culture reagents were purchased from Invitrogen. HeLa and U2OS cells were cultured in Dulbecco's modified eagle medium (high glucose with Glutamax) containing 10% fetal calf serum and penicillin–streptomycin. *C. trachomatis* LGV2 serovars were propagated in HeLa cells and stored at −80 °C in sucrose–phosphate–glutamate buffer^47^.

### Sample preparation

For preparation of *Chlamydia*-infected cells for cryo-electron tomography, HeLa or U2OS cells were seeded into well plates and infected with *C. trachomatis* LGV2 (MOI 5)^18^. The following day adherent cells were seeded onto 200 mesh gold grids (R3.5/1; Quantifoil Micro Tools, Jena, Germany) at a density of one or two cells per grid square. At ∼48 h post infection, EM grids with or without host cells were introduced to the tissue culture dish and incubated with the newly released elementary body progeny (15 min–1 h at 37 °C). Grids were removed and rinsed in Hank's buffered salt solution. Four microlitres of BSA-coated gold (Sigma) was added to the grid before it was plunge frozen into liquid ethane (Vitrobot Mark IV, FEI).

*Salmonella enterica* serovar Typhimurium strain ΔaraBAD1167::hilA+ Δtar-flhD2039 ΔminCDE::tetRA was grown in Luria-Bertani broth (LB) overnight at 37 °C with aeration. The following day, 50 μl of the overnight was subcultured into 5 ml LB and supplemented with 0.1% L-arabinose and 10 μg ml^−1^ tetracycline. After 5 h, rods and cellular debris were isolated by centrifugation at 2,000*g* for 10 minutes at 4 °C. Minicells were isolated from the supernatant by centrifugation at 15,300*g* for 10 min at 4 °C. The minicell pellet was resuspended in BSA-coated colloidal gold (Sigma) and 4 μl of the suspension was added to Quantifoil grids before being plunge frozen into liquid ethane.

### Cryo-electron tomography

For cryo-electron tomography, single-axis tilt series were collected with SerialEM^48^ on a 300-kV Tecnai Polara electron microscope equipped with a K2 Summit direct electron detector and Quantum energy filter (Gatan). An energy window of 20 eV was used for recording zero-loss images and samples were maintained at liquid nitrogen temperatures. Images were recorded over a range of −60° to +45° with 3° increment at a 10-μm defocus and 5.4 Å pixel size. Tilt images were collected as eight sub-frames in electron-counting mode with a dose rate of six to seven electrons per pixel per second yielding a total dose of 1.5 electrons per Å^2^ for each tilt image and a cumulative dose of 54 electrons per Å^2^ for each tilt series. Sub-frames were aligned in Digital Micrograph before being incorporated into the final image stack in SerialEM. Tilt images were aligned in IMOD^49^ using 10-nm gold fiducials and CTF correction was applied before tomogram reconstruction by weighted back-projection^50^. Nonlinear anisotropic filtering was applied to tomograms for sub-tomogram identification and selection^51^.

### Image processing

Sub-tomogram positions were manually selected in IMOD and extracted from raw tomograms using the dtcrop function in Dynamo^52^. Initial alignment was performed manually with the Dynamo gallery. Further sub-tomogram alignment and averaging was conducted in Dynamo with a modification of splitting particles into two independent data sets for the final resolution measurement. First, asymmetric averages were generated by aligning the outer and the inner membranes separately. Six-fold rotational symmetry was detected in the sorting platform by rotational correlation (Supplementary Fig. 6) and imposed for further refinement. Twelve-fold symmetry was applied to the *Chlamydia* and *Salmonella* basal body to increase the signal-to-noise ratio of the final structures, in accordance with symmetry determined by single-particle analysis^3^. Note that applying axial symmetry does not change interpretation of the data. After convergence, the final structures from each data set were merged at the midpoint of the volume and low-pass filtered to the detected resolution. Classification by multi-reference alignment was performed by first generating 5 or 10 initial references by adding 10% Gaussian noise to a global average (made from all ∼1,200 sub-tomograms) yielding a signal-to-noise ratio of 0.9. Starting from an initial global alignment, sub-tomograms were iteratively aligned to each reference using a bottle-like alignment mask that encompassed the basal body, inner membrane and sorting platform–ATPase. The maximum angular range used for searching was 6° and the maximum shift allowed was 5 voxels. After each round of alignment, sub-tomograms contribute once to the reference to which it had the highest correlation coefficient. Class averages were produced after convergence when the sub-tomograms no longer changed class memberships. Classification by principal component analysis and k-means did not cleanly separate sub-tomograms into groups with and without the sorting platform.

### Segmentation, surface renderings and measurements

Automatic segmentation of membranes was performed with TomoSegMem^53^ and refined manually in Amira (FEI Visualization Sciences Group, Massachusetts, USA). Segmentation of actin filaments was conducted in IMOD. The EM Package for Amira was used to map individual T3SS models into their corresponding positions in cryo-electron tomograms^54^. Multiple sequence alignments were produced by ClustalW Omega^55^ and graphically illustrated with BOXSHADE. Distances between the bacterial outer membrane and host membrane were measured in individual tomographic slices where a T3SS needle contact was clearly resolved. Measurements were collected in IMOD by drawing a line along the T3SS needle from the centre of the outer membrane to the centre of the plasma membrane and calculating the number of pixels the line spanned. For non-T3SS-mediated contacts, measurements were collected from tomographic slices that depicted a constant spacing between the non-T3SS containing bacterial hemisphere and the host plasma membrane. A line perpendicular to the apposed membranes was drawn in IMOD and converted to distance in pixels.

## Additional information

**Accession codes:** Representative sub-tomogram averages have been deposited in the EMDB (accession codes EMD-3216 and EMD-3217) and tomograms into EMPIAR (EMPIAR-10047 and EMPIAR-10048).

**How to cite this article:** Nans, A. *et al.* Structure of a bacterial type III secretion system in contact with a host membrane *in situ*. *Nat. Commun.* 6:10114 doi: 10.1038/ncomms10114 (2015).

## Supplementary Material

Supplementary Information Supplementary Figures 1-7

Supplementary Movie 1 Transition of the Chlamydia type III secretion system from the host-free to host-contact state. Basal body compaction and sorting platform-ATPase complex stabilization visualized as a linear morph between the host-free and host-active sub-tomogram averages. The video was created using the iMorph plugin for Fiji.
