# Supplementary material for: Structure of a bacterial type III secretion system in contact with a host membrane in situ
Source: Nat Commun. 2015 Dec 11;6:10114. doi: 10.1038/ncomms10114 (PMC4682100; doi:10.1038/ncomms10114)
Supplement: Supplementary Information — Supplementary Figures 1-7 [file ncomms10114-s1.pdf]

a

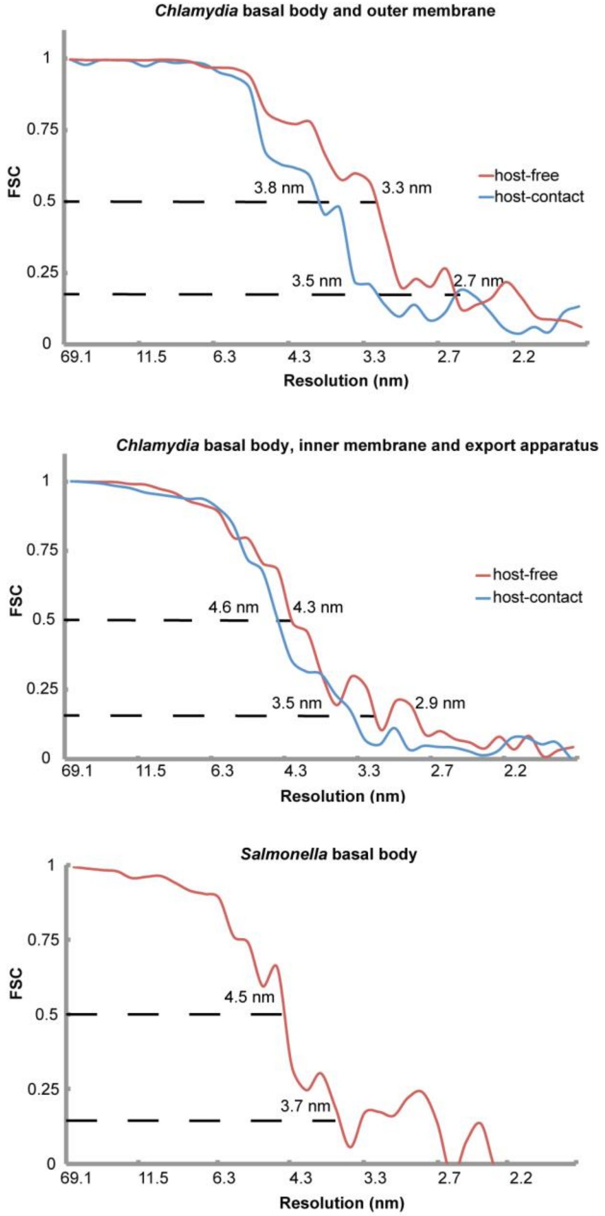

b

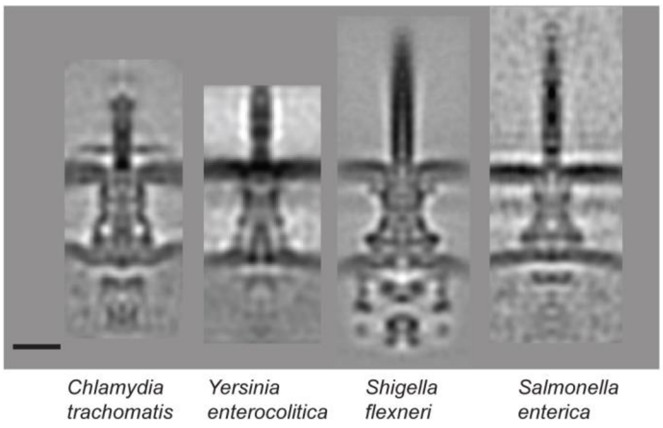

Chlamydia trachomatis *Yersinia enterocolitica* *Shigella flexneri* *Salmonella enterica*

Supplementary Figure 1. Fourier shell correlation curves for sub-tomogram averages and comparisons to other published *in situ* T3SS structures. a, Resolution estimates after applying Fourier shell correlation to independently processed half-data sets (gold-standard procedure, 0.5/0.143 criterion are both indicated. (upper graph) FSC curve for the host-free (red, 3.3 nm) and host-contact T3SS (blue, 3.8 nm) assuming 12-fold symmetry and using a mask to align the basal body and outer membrane. (middle graph) FSC curve for the host-free (red, 4.3 nm) and host-contact (blue, 4.6 nm) T3SS assuming 6-fold symmetry and using a mask to align the basal body, inner membrane, and sorting platform-ATPase in the cytosol. (lower graph) FSC curve for host-free *Salmonella* T3SS basal body assuming 12-fold symmetry. **b**, Side-by-side comparison of the T3SS from host-free *Chlamydia* to T3SS from the bacterial envelope of *Yersinia enterocolitica* (EMD-5694), *Shigella flexneri* (EMD-2667), and *Salmonella enterica* (Fig. 1d) mini-cells.

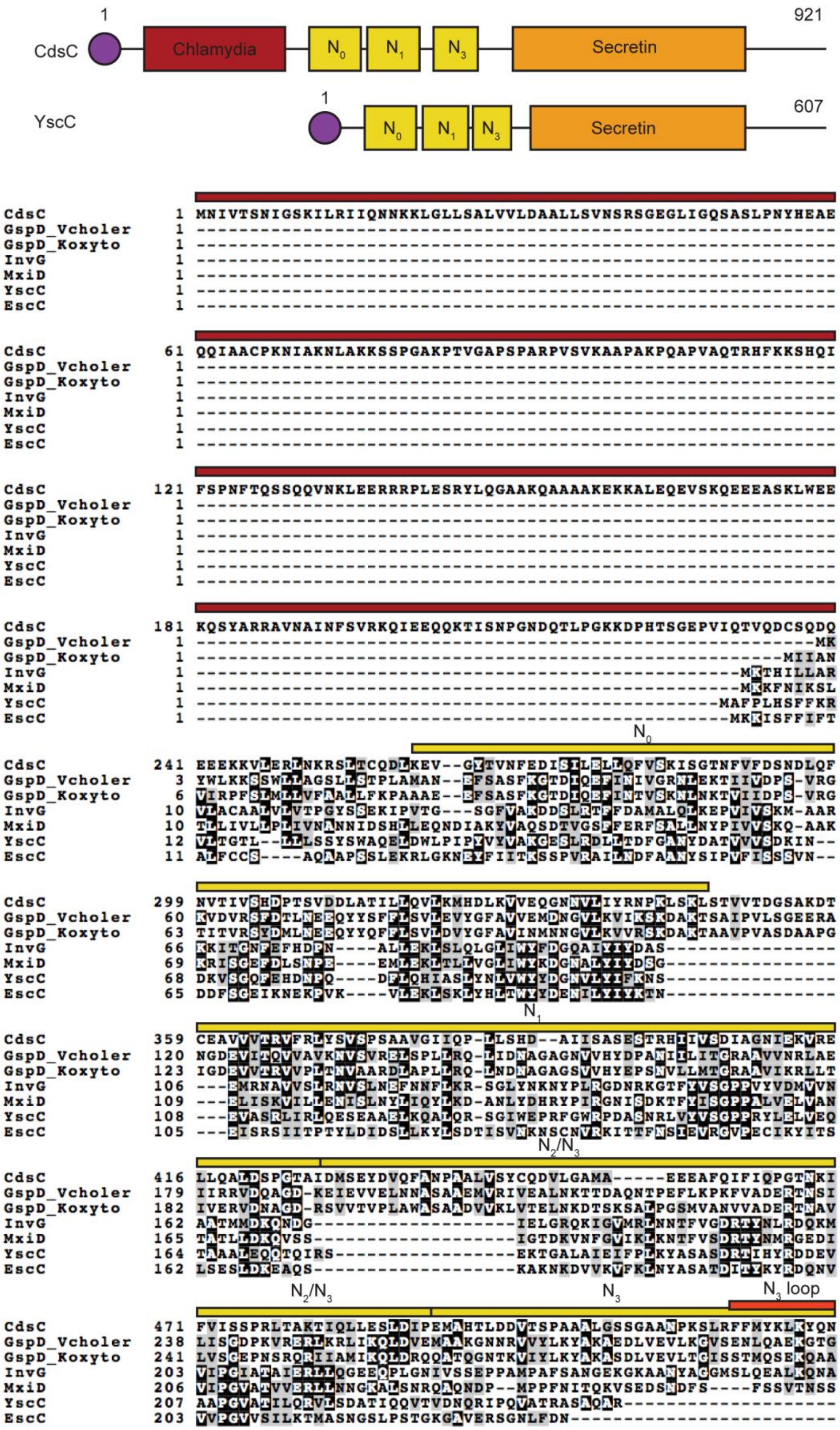

CdsC
1
Chlamydia
N_0
N_1
N_3
Secretin
921
YscC
1
N_0
N_1
N_3
Secretin
607
CdsC
GspD_Vcholer
GspD_Koxyto
InvG
MxiD
YscC
EscC
N_0
N_1
N_2/N_3
N_3
N_3 loop

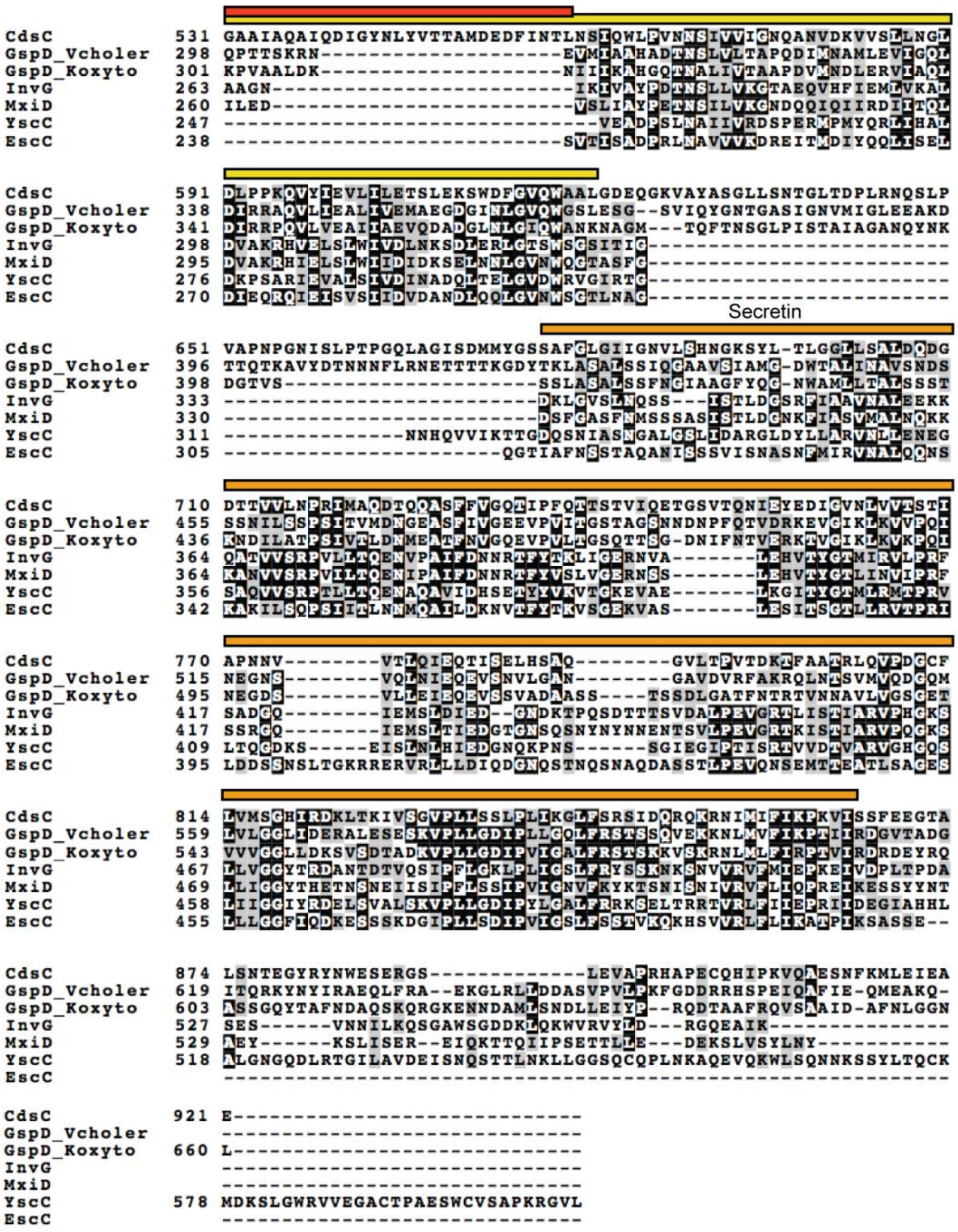

Supplementary Figure 2. Domain architecture of CdsC and YscC and the corresponding multiple sequence alignment of type II and type III secretin homologues (GspD and SctC). The *Chlamydia* secretin, CdsC, contains an N-terminal domain of 250 amino acids (dark red) in addition to the classical N-domains (yellow) found in other type II or type III secretins. CdsC

also uniquely contains type-II specific insertions throughout the N-domains, in the secretin domain (orange) and in the N3 loop (red). The predicted signal-peptide is also indicated (purple). Multiple-sequence alignments were produced by ClustalW Omega and graphically illustrated with BOXSHADE. Identical residues are shaded in black and similar residues in grey. Colored annotations in the sequence alignment correspond to the domains in the secretin schematic. Accession numbers are listed for each bacterial species. CdsC, *C. trachomatis* (M9UG25); GspD, *V. cholera* (C3LSG0); GspD, *K. oxytoca* (A0A068H9C0); InvG, *S. enterica* (P35672); MxiD, *S. flexneri* (Q04641); YscC, *Y. enterocolitica* (Q93KT1); EscC, enteropathogenic *E. coli* (B7UMB3).

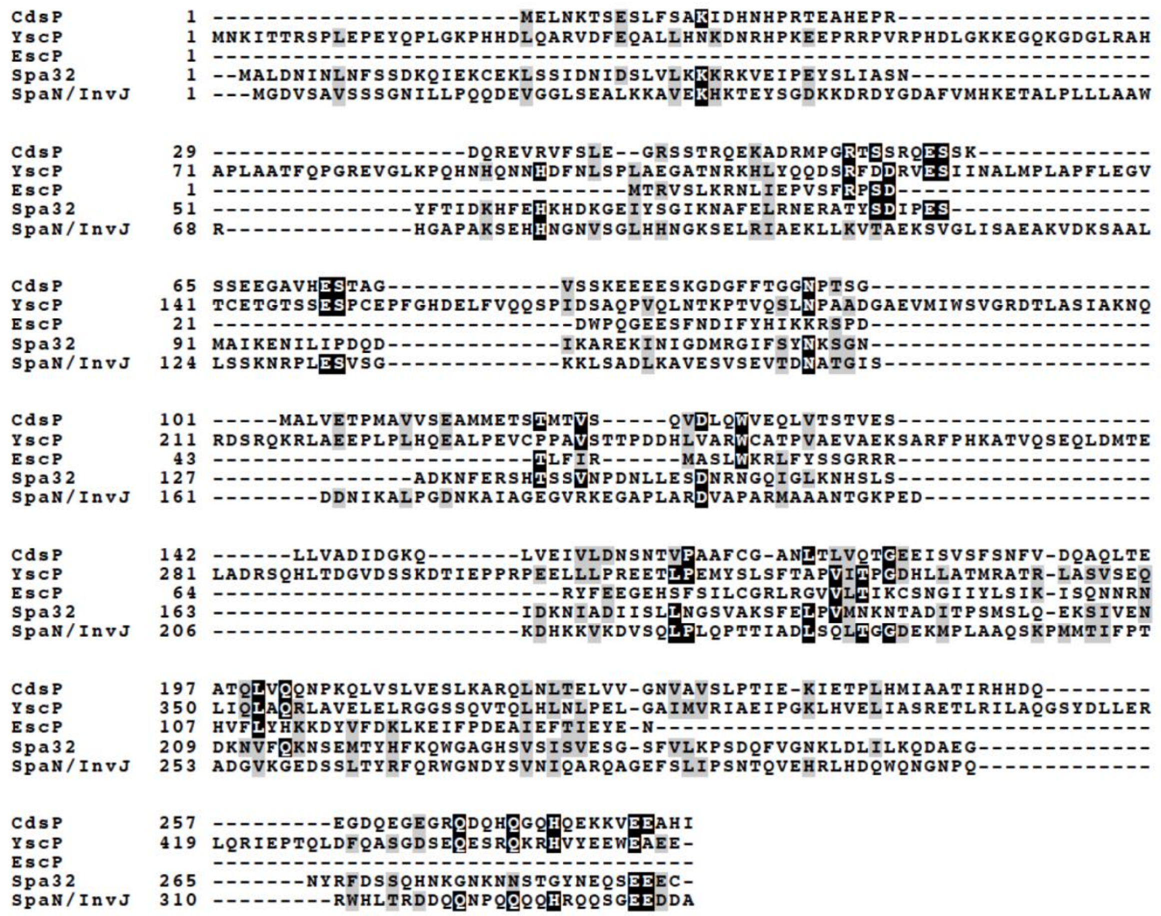

Supplementary Figure 3. Multiple sequence alignment of tape measure protein SctP. The tape measure protein SctP regulates the needle length in T3SS-containing bacterial pathogens. Residue length and accession numbers are listed for each bacterial species. CdsP, *C. trachomatis* (283, M9UDI3); YscP, *Y. enterocolitica* (453, Q93KT6); EscP, enteropathogenic *E. coli* (138, B7UMA4); Spa32, *S. flexneri* (292, D2AJK8); SpaN/InvJ, *S. enterica* (336, P40613). Multiple sequence alignments were produced by ClustalW Omega and graphically illustrated with BOXSHADE. Identical and similar residues are shaded in black and grey respectively.

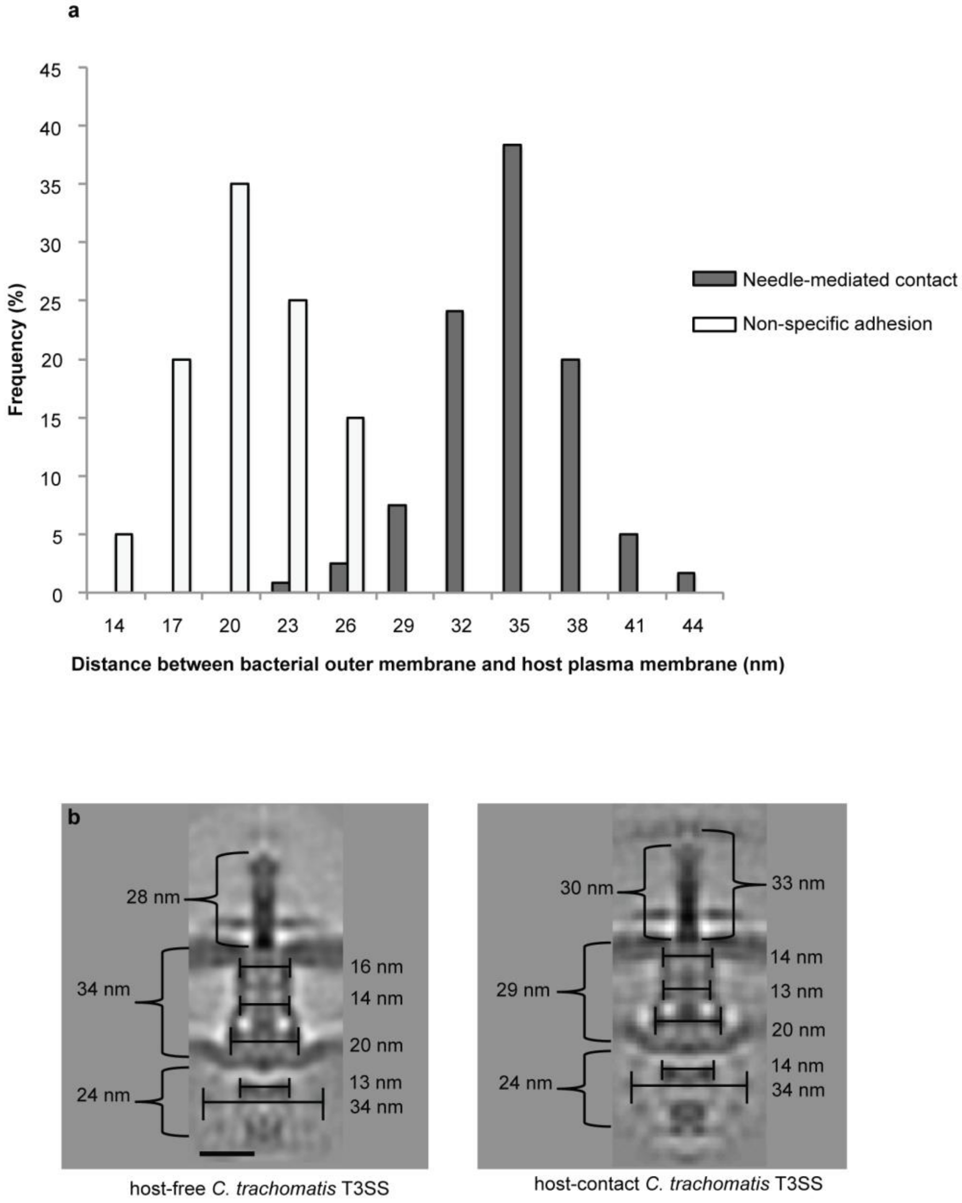

Supplementary Figure 4. Distance between bacterial outer membrane and target host membrane. a, Histogram of length measurements for needle-mediated contact (average 33 ± 4 nm; range 22-44 nm; n =120) and non-specific adhesion (average 20 ± 3 nm; range 14-25 nm; n = 20). Measurements in both cases were recorded manually from representative tomogram slices in IMOD **b**, Dimension comparisons for host-free and host-contact *Chlamydia* T3SSs.

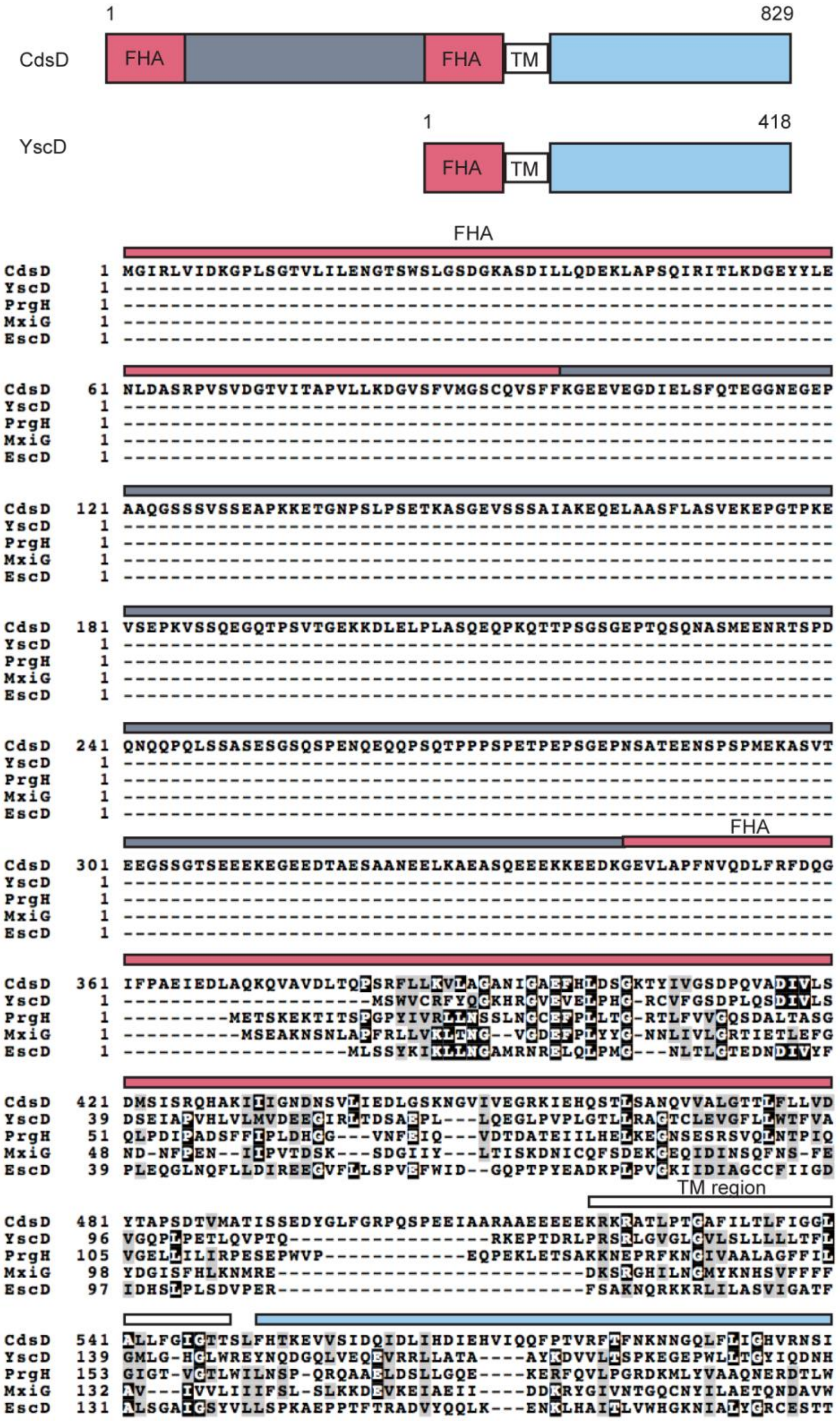

1
829
CdsD
FHA
FHA
TM
1
418
YscD
FHA
TM
FHA
FHA
TM region
CdsD
YscD
PrgH
MxiG
EscD

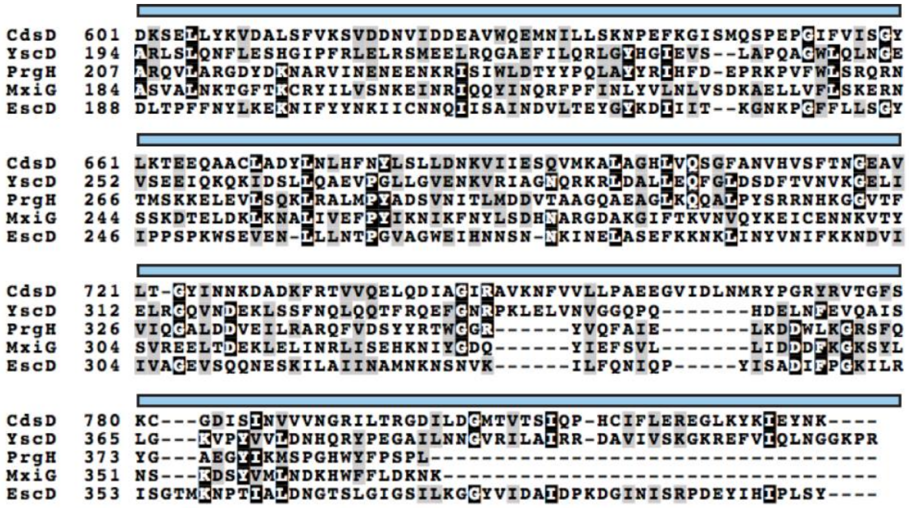

Supplementary Figure 5. Domain architecture of major inner membrane proteins CdsD and YscD and multiple sequence alignment of SctD homologues. The *Chlamydia* inner membrane protein, CdsD, contains an additional N-terminal segment of 360 residues (pink and grey) including an extra cytoplasmic FHA domain. Colored annotations in the sequence alignment correspond to the domains in the CdsD/YscD schematic. Accession numbers are listed for each bacterial species. CdsD, *C. trachomatis* (M9UG17); YscC. *Y. enterocolitica* (Q01245); PrgH, *S. enterica* (P41783). MxiG, *S. flexneri* (P0A221); EscD, enteropathogenic *E. coli* (B7UM96). Multiple sequence alignments were produced by ClustalW Omega and graphically illustrated with BOXSHADE. Identical and similar residues are shaded in black and grey respectively.

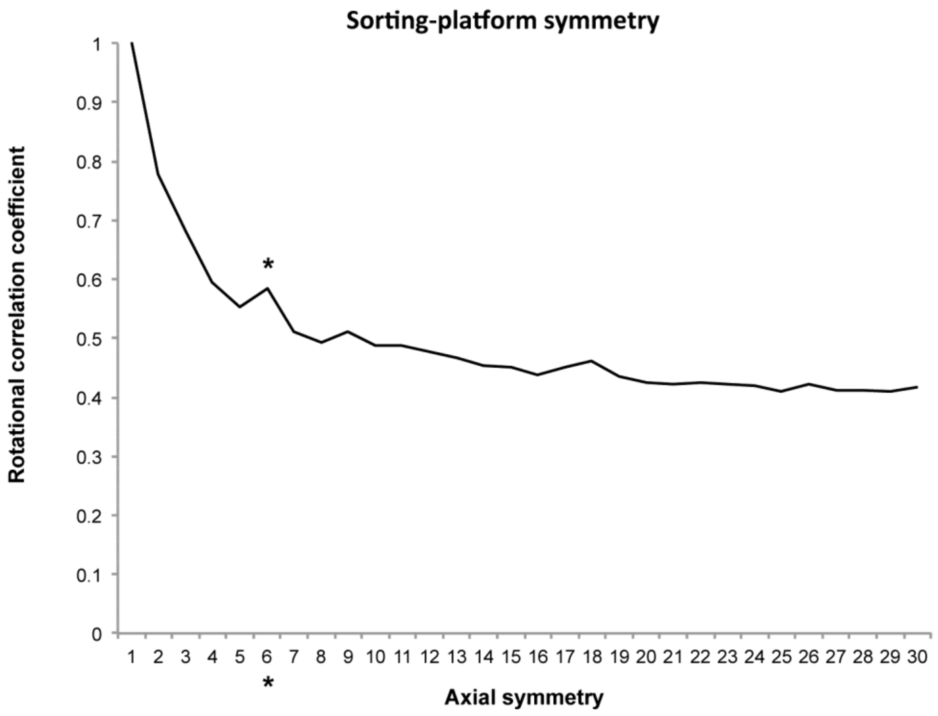

Supplementary Figure 6. Symmetry analysis of *Chlamydia* T3SS sorting platform. To detect symmetry in the sorting platform, a masked asymmetric average was compared to a similarly masked average that had been rotated axially by intervals corresponding to rotational symmetries from c1 to c30. The cross-correlation coefficient was plotted as a function of the corresponding symmetry. Peaks along the cross-correlation coefficient plot will be observed when a rotated average exhibits a high degree of similarity with the unrotated average, demonstrating rotational symmetry.

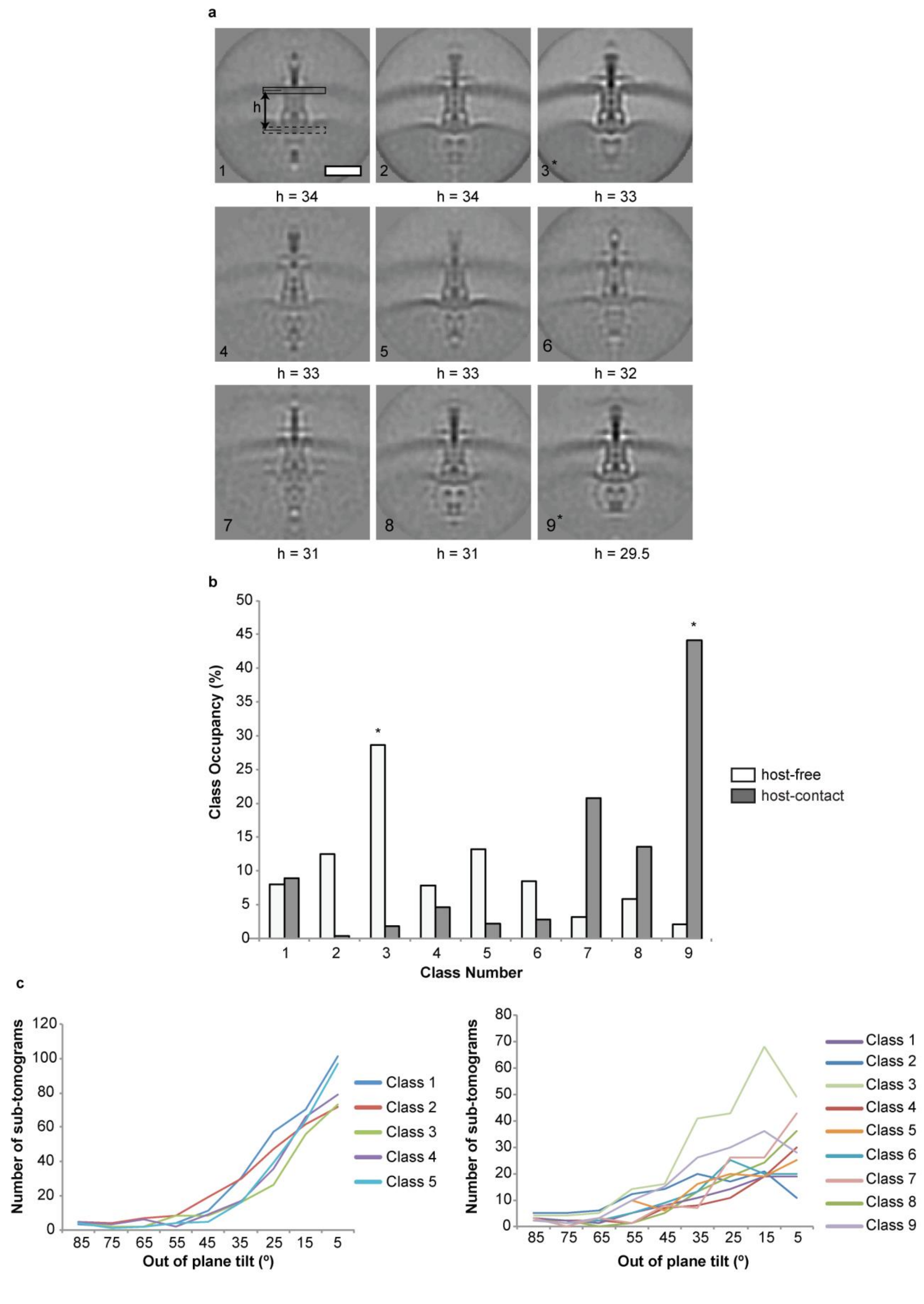
a
h
1
h = 34
2
h = 34
3*
h = 33
4
h = 33
5
h = 33
6
h = 32
7
h = 31
8
h = 31
9*
h = 29.5
b
Class Occupancy (%)
50
45
40
35
30
25
20
15
10
5
0
*
*
host-free
host-contact
1 2 3 4 5 6 7 8 9
Class Number
c
Number of sub-tomograms
120
100
80
60
40
20
0
85 75 65 55 45 35 25 15 5
Out of plane tilt (º)
Class 1
Class 2
Class 3
Class 4
Class 5
Number of sub-tomograms
80
70
60
50
40
30
20
10
0
85 75 65 55 45 35 25 15 5
Out of plane tilt (º)
Class 1
Class 2
Class 3
Class 4
Class 5
Class 6
Class 7
Class 8
Class 9

Supplementary Figure 7. Multi-reference alignment classification of *Chlamydia* sub-tomograms. Sub-tomograms were initially separated into 10 classes by multi-reference alignment. One of the 10 class averages contained mostly noise and thus has been eliminated. **a**, Central slices through 9 class averages of *Chlamydia* T3SSs. The class averages are sorted by basal body height (h), which varies from 34 to 29.5 nm. **b**, Distribution of host-free and host-contact *Chlamydia* T3SSs across the 9 classes depicted in (a). The most populated classes are marked with an asterisk. Host-free T3SSs were mainly present in classes with elongated basal bodies (classes 1-6). Host-contact T3SSs were mainly present in classes with short, compact basal bodies (classes 7-9). **c**, Out of plane tilt of all sub-tomograms using MRA with 5 classes (left, and as shown in Fig. 3) and with 9 classes (right, as shown in (a)). There is no predominant bias in the distribution of particles into classes based on their out of plane tilt angle. Scale bar, 30 nm.
